# Impact of inspiration level on lung nodule volumetry

**DOI:** 10.1016/j.redii.2024.100043

**Published:** 2024-03-12

**Authors:** Arnaud Halter, Aïssam Labani, Catherine Roy, Mickaël Ohana

**Affiliations:** Diagnostic Imagery Department, Nouvel Hôpital Civil (NHC), Hôpitaux Universitaires de Strasbourg, 17, rue de la Porte-de-l'Hôpital, 67000 Strasbourg, France

**Keywords:** Nodule, Size, Volumetry, Inspiratory level, Residual volume

## 1 Introduction

Lung nodule volumetry is a validated tool to manage indeterminate pulmonary nodules [1] with volume doubling times calculation [2]. While its better reproducibility over uni- or bidimensional electronic caliper–based diameter measurements in assessing nodules sizes is validated [3], the impact of patient-related technical factors such as inspiratory level on its reliability is debated, with inconsistent results in the literature on the way it could influence its results [4].

The primary objective of our study was to investigate the effect of inspiratory level on lung nodules volumetry. The secondary objective was to query the amplitude of respiratory effort and nodule-specific characteristics as predictive factors of nodule volume variations.

## 2 Material and methods

This monocentric retrospective observational study was conducted during the year 2022 in a French university hospital. Patients considered for inclusion underwent unenhanced chest CT including paired acquisitions: an inspiratory CT-scan, immediately followed by an end-expiratory CT-scan, acquired between 2015 and 01–12 and 2021–12–22.

A total of 403 examinations were thus consulted by a single operator (one radiologist with 4 years of training in CT) for preselection and patients were included in our study if they met the following inclusion criteria:

- presence of one or more nodule, as defined by the *Fleischner Society: Glossary of Terms for Thoracic Imaging* [5]
- with a greater diameter (on inspiratory CT-scan measured in native axial plane) of at least 6 mm.

Typical and atypical perifissural lung nodules presumed of lymphatic nature, calcified nodules, excavated nodules and nodules issued from exams judged to be technically insufficient/unconform were excluded.

For each included nodule were listed its size (greater diameter on inspiratory CT-scan measured in native axial plane), morphology (solid, ground-glass, part-solid), lobar topography, closeness to the pleura (subpleural or not) and the regularity of its margins (regular/irregular).

CT were acquired on 320-row scanners, with identical technical parameters (collimation : 0,5 × 80 mm*,* gantry rotation time: 0,275 s*,* pitch : 0.813, slice thickness/reconstruction increment: 1 mm/0,8 mm*).* Raw-image data was worked-off on a mediastinal-window using iterative reconstruction (AID3RD) or deep-learning reconstruction (AiCE).

Lung nodule semi-automated segmentation and lung parenchyma segmentation was performed by the same radiologist using Vitrea's « CT Lung Nodule Analysis » software (version 6.3, Canon Medical Informatics, Minneapolis, Minnesota, USA), with careful manual edition, if judged necessary by the operator.

Finally, nodules and lung volumes variations between inspiratory and expiratory acquisitions were calculated for each pair of exams as followed:

$$volume\;variation\;\left(\%\right)=\frac{\;V_{BMI}\;-\;V_{R}^{\;}\;}{V_{BMI}}\times \;100$$

Where $V_{BMI}$ stands for a nodule of interest/lung volume on inspiratory CT – i.e., at blocked maximum inspiration – and $V_{R}\;$stands for a nodule of interest/lung volume in expiratory CT – i.e., at residual lung volume.

A paired Student's *t*-test allowed the comparison of changes in nodule volumes between inspiratory and expiratory acquisitions while a multiple linear regression model was used to identify predictive variables of the effect of inspiratory level on nodule volumes.

## 3 Results

**Fig. 1:**
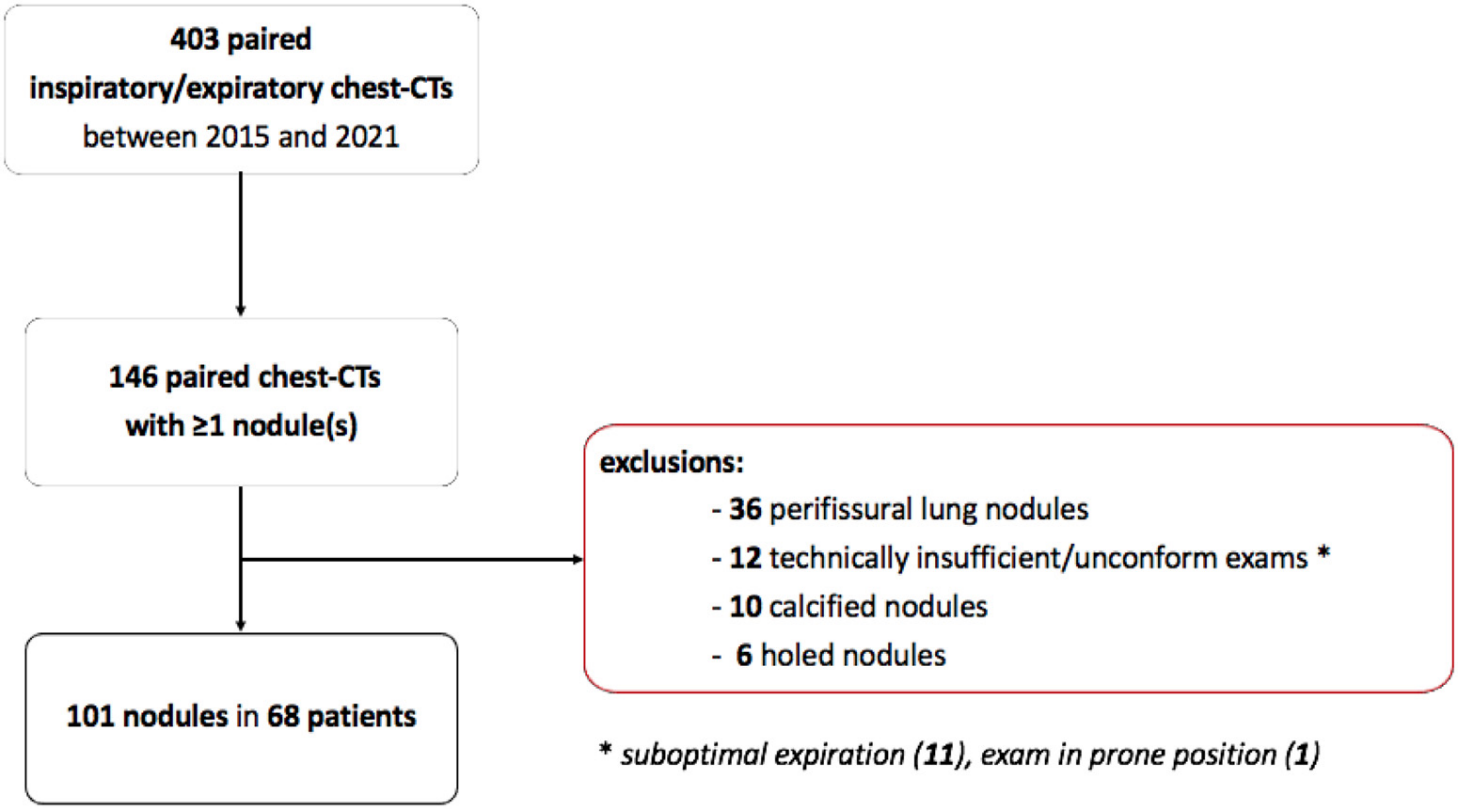
Study population selection flowchart.

A total of 101 nodules in 68 patients were finally included and analyzed (Fig. 1& Table 1); their inspiration/expiration differential volume variations were found to be significant (*p* *<* 0.001); an increased size was shown at residual volume for most of them (75/101, 74 %). This result is consistent with those of Jin Mo Goo et al. [6] and Moser et al. [7] who reported a volume increase of respectively 85 % (28/33) and 67 % (59/88) of the nodules included in their studies.

**Table 1:** Nodules characteristics.

	Total (*n* = 101)
*Lobar topography* Right upper lobe Right inferior lobe Left inferior lobe Left upper lobe Right middle lobe	31 26 23 19 2
*Morphology* Dolid Ground-glass Part-solid	82 14 5
*Nodule margin* Regular Irregular	80 21
*Location* Subpleural Central	37 64
*Size (mm : mean - σ)*	9 - 3,9 [6–28]
*Volume (mm^3^ : mean - σ)* Inspiration Expiration	530 - 696 545 - 683

**Fig. 2:**
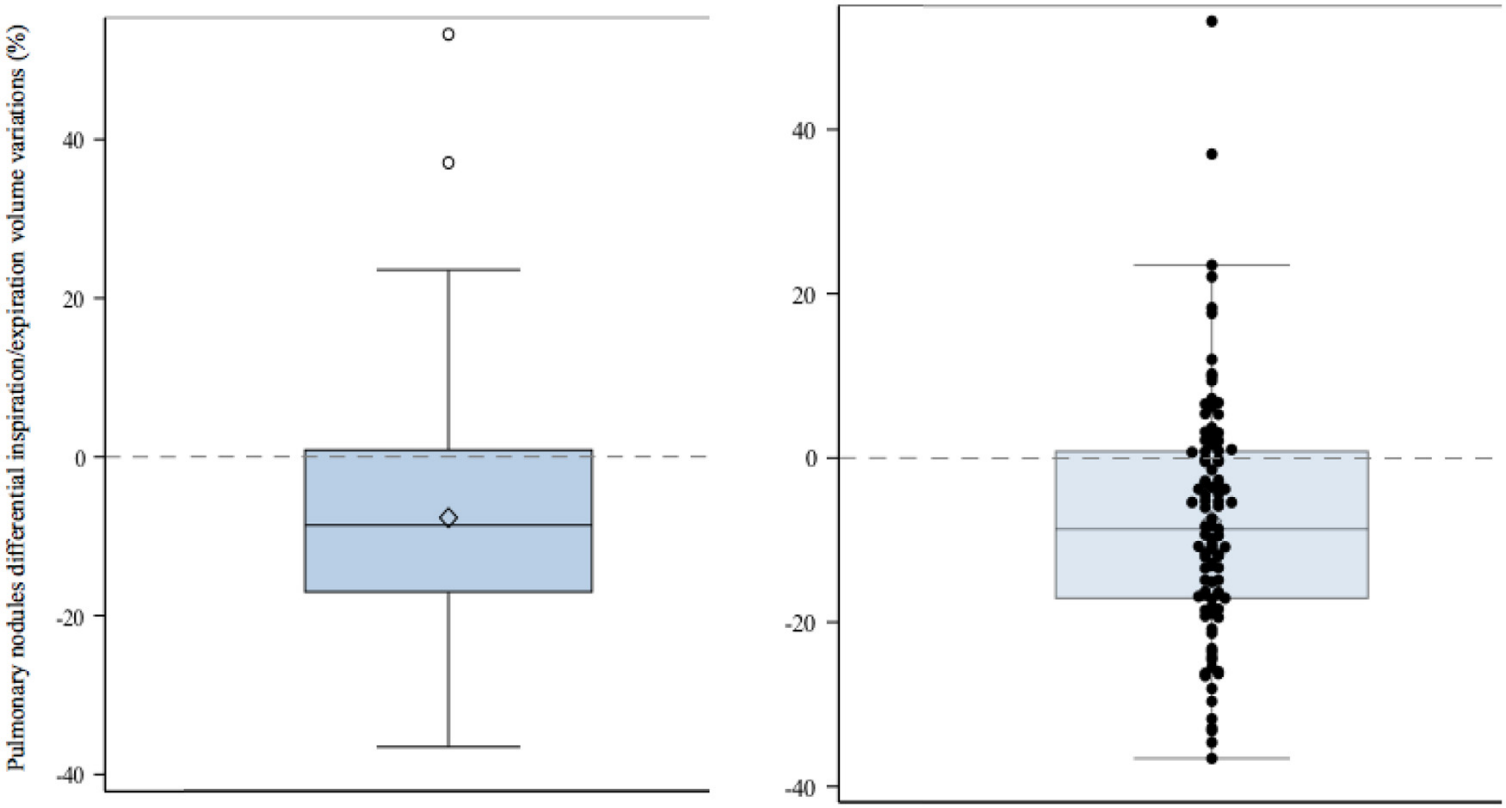
Pulmonary nodules differential inspiration/expiration volume variations’ value distribution illustrated by a box plot (left) with a superposed scatter plot for each nodule's volume variation value (right).

The average nodule volume variation of 8 % (95 % CI [4.77; 10.63], Fig. 2) in our study suggests a mild influence of inspiration level over lung nodules volumetry when compared to other technical parameters like measurement tools: Ashraf et al. [8] reported a volume variation exceeding 25 % for more than 80 % of nodules measured with two different segmentation algorithms of the same volumetry software.

No correlation was found in regression analysis between nodule-specific characteristics (lobar topography, morphology, nodule margin, closeness to the pleura) or the amplitude of respiratory effort – i.e., differential inspiration/expiration lungs volume variations, a result consistent with those of Petkovska et al. [9] and Moser et al. [7] – and nodule volume variations. It is noticeable that the amplitude of respiratory effort we observed (l45 %l, [−23 % ; −61 %]) was far greater than what could be expected in clinical practice (+2 % [−19 % ; +26 %] in de Hoop et al. [10]); thus limiting the extrapolation of our results.

The use of volume variations might also be pointed as a limit to our results given the size range of included nodules. However, when applying the mean nodule size variation in our study (8 %) to even the biggest nodules, the corresponding diameter measurement differential was inframillimetric.

Additional main limits of our study include its monocentric retrospective structure and an unique operator.

## 4 Conclusion

Inspiration level significantly affects lung nodules volumetry with a trend towards increase at residual volume. While present, this effect is modest and unpredictable, suggesting questionable involvement in clinical practice.

## Internal review board approval and consent

This study was approved by the ethics committee of our institution (University Hospitals Strasbourg, France). Written consent was not required for this retrospective study.

## Research data

The data that support the findings of this study are not openly available due to reasons of sensitivity and are available from the corresponding author upon reasonable request.

## CRediT authorship contribution statement

**Arnaud Halter:** Formal analysis, Investigation, Writing – original draft, Visualization. **Aïssam Labani:** Conceptualization, Supervision, Resources. **Catherine Roy:** Supervision. **Mickaël Ohana:** Conceptualization, Methodology, Validation, Resources, Writing – review & editing, Supervision.

## Declaration of competing interest

The authors declare the following financial interests/personal relationships which may be considered as potential competing interests:

Mickaël Ohana, MD, PhD; Editor-in-Chief of the journal is cited as author.
